# Two new species of *Fluminicola* (Caenogastropoda, Lithoglyphidae) from southwest Oregon, USA, and a range extension for *F. multifarius*

**DOI:** 10.3897/zookeys.679.13472

**Published:** 2017-06-08

**Authors:** Robert Hershler, Hsiu-Ping Liu, Niko Hubbart

**Affiliations:** 1 Department of Invertebrate Zoology, Smithsonian Institution, Washington, D.C. 20013-7012, USA; 2 Department of Biology, Metropolitan State University of Denver, Denver, CO 80217, USA

**Keywords:** Caenogastropoda, Truncatelloidea, freshwater, North America, systematics, morphology, mitochondrial DNA

## Abstract

We describe two new species of pebblesnails (Lithoglyphidae: *Fluminicola*) from southwest Oregon based on morphologic and mitochondrial DNA (COI, cytB) evidence. *Fluminicola umpquaensis* **sp. n.**, which had been traditionally identified as *F. virens* prior to the recent restriction of the latter to the lower Columbia River drainage, lives in lotic habitats in the Umpqua River basin. This species is readily distinguished from closely related *F. gustafsoni* and *F. virens* by shell and anatomical characters, and by its mtDNA sequences (divergence >3.6% for both genes). *Fluminicola fresti* **sp. n.** ranges among lotic habitats in the North Umpqua River basin, and in the upper Rogue River drainage north of Little Butte Creek. This species differs from other congeners by >9.1% for both genes and is distinguished from closely similar and geographically proximal *F. multifarius* by several anatomical characters. Additionally, new records are provided for *F. multifarius* from the upper Rogue River basin south of Little Butte Creek, which extend the geographic range of this species about 80 km northward from the Sacramento River headwater region. This continues a recent series of taxonomic papers on the poorly known and little studied pebblesnail fauna of the vast Pacific watershed from northern California to southern British Columbia.

## Introduction

*Fluminicola* (Truncatelloidea, Lithoglyphidae) is a western North American genus of small freshwater gastropods with globose to conical shells, commonly known as pebblesnails, which is distributed in diverse habitats ranging from small seeps to spring-influenced lacustrine reaches to large rivers. Both morphological (Hershler and Frest 1996) and molecular (Hershler and Liu 2012) evidence suggest that the 25 currently recognized *Fluminicola* species belong to two evolutionarily distinct lineages. One of the lineages contains two species in the Columbia River basin – *F. gustafsoni* Hershler & Liu, 2012; *F. virens* (Lea, 1838) – while the other (containing the remaining congeners) is distributed in this drainage and also the Great Basin, upper Colorado River basin, and Sacramento River basin. *Fluminicola* continues to be recognized as a non-monophyletic genus pending clarification of the phylogenetic relationships of its poorly known and possibly extinct type species, *F. nuttallianus* (Lea, 1838).

The broad geographic range of *Fluminicola* includes most of the Pacific Coastal watershed from northern California to southern British Columbia. Pebblesnail populations are scattered throughout much of this huge area, yet are currently undescribed aside from six species in the Columbia River basin (Hershler and Frest 1996, Hershler and Liu 2012, Liu et al. 2013) and 14 species in the Sacramento River basin (Hershler et al. 2007). This taxonomic knowledge gap is hampering efforts by the conservation community to obtain legal protection for pebblesnails (e.g., USFWS 2012), which are groundwater-dependent and threatened by various anthropogenic activities.

The *Fluminicola* fauna of southwestern Oregon includes a relatively large pebblesnail in the Umpqua River basin that was traditionally identified as *F. virens* prior to the restriction of the latter to the lower Columbia River drainage (Hershler and Frest 1996), and numerous unstudied populations of smaller pebblesnails in the upper reaches of both the Rogue and Umpqua River basins that were recently reported in grey literature (Frest and Johannes 1999, 2000, 2004, 2005). Herein we utilize DNA sequences from two mitochondrial genes in delineating the *Fluminicola* species in the Rogue-Umpqua basins. This continues our recent series of integrative taxonomic studies of the pebblesnails of the Pacific Coastal drainages (Hershler et al. 2007, Hershler and Liu 2012, Liu et al. 2013).

## Methods

We sequenced specimens from 35 sites in the Rogue-Umpqua basins, including eight localities containing the large pebblesnail resembling *F. virens* and 27 localities containing smaller pebblesnails. Thirty-two of these localities were sampled by RH and HPL during September 2015 specifically for this project. Specimens were collected by hand or with a small sieve and preserved in 90% (non-denatured) ethanol in the field. Vouchers were deposited in the Smithsonian Institution’s National Museum of Natural History (USNM) collection. Other relevant material from the USNM and the Bell Museum of Natural History (BellMNH) was also examined during the course of this study.

A total of 155 and 146 sequences of cytochrome c oxidase subunit I (COI) and cytochrome B (cytB), respectively were obtained from 161 analyzed Rogue-Umpqua pebblesnails. Genomic DNA was extracted from entire snails using a CTAB protocol (Bucklin 1992); each specimen was analyzed for mtDNA individually. LCO1490 and HCO2198 (Folmer et al. 1994) were used to amplify a 708 base pair (bp) fragment of COI; cytB427F (5’TGA GGK GCN ACT GTT ATT ACT AA3’) and cytB1049R (5’GTG AAA ACT TGS CCR ATT TGC TC3’) were used to amplify a 644 bp fragment of the cytB gene. The cytB427F and cytB1049R primers were designed based on conserved regions of cytB in an alignment using previously published sequences from *Oncomelania hupensis* (Gredler) (NC13073) and *Potamopyrgus antipodarum* (Gray) (GQ996433). Amplification conditions and sequencing of amplified polymerase chain reaction product methods were those of Liu et al. (2013). Sequences were determined for both strands and then edited and aligned using Sequencher™ version 5.4.1 (Gene Codes Corporation, Ann Arbor, MI). Our analysis of the COI dataset also included 35 previously published sequences from 23 congeners, two taxonomically indeterminate *Fluminicola* lineages from the Sacramento River basin (*Fluminicola* sp. A, *Fluminicola* sp. B, Hershler et al. 2007), and representatives of two other North American lithogyphid genera (*Somatogyrus*, *Taylorconcha*). Trees were rooted with *Pristinicola hemphilli* (Pilsbry) (Hydrobiidae). The cytB dataset also included 34 previously published sequences from 22 *Fluminicola* species (a cytB sequence is not available for *F. gustafsoni*) and the two taxonomically indeterminate *Fluminicola* lineages. Given that cytB sequences are not available for other North American lithoglyphid genera, we used basally positioned *F. virens* to root the trees (Hershler et al. 2007, Hershler and Liu 2012). In order to generate easily readable topologies, one example of each detected haplotype was used in the phylogenetic analyses, which were performed separately for the COI and cytB datasets. Sample codes, locality and voucher details, and GenBank accession numbers for the sequences used in the molecular phylogenetic analyses are in Suppl. material 1.

Genetic distances were calculated using MEGA7 (Kumar et al. 2016), with standard errors estimated by 1,000 bootstrap replications with pairwise deletion of missing data. MrModeltest v. 2.3 (Nylander 2004) selected the GTR + I + G model parameters as the best fit for both the COI and cytB datasets (using the Akaike Information Criterion). Phylogenetic analyses were performed using three different methodologies – maximum parsimony (MP), maximum likelihood (ML), and Bayesian inference. The MP and ML analyses were performed using PAUP* v. 4.0b10 (Swofford 2003) and the Bayesian analyses were conducted using MrBayes v. 3.2.6 (Ronquist and Huelsenbeck 2003). The MP analysis was conducted with equal weighting, using the heuristic search option with tree bisection reconnection branch-swapping and 100 random additions. Nodal support was evaluated by 10,000 bootstrap replicates. The ML analysis was performed using the GTR + I + G model. The optimized parameter values for COI were base frequencies of A = 0.3097, T = 0.3952, C = 0.1612, G = 0.1339; shape of gamma distribution = 1.3430; proportion of invariant sites = 0.5912. The optimized parameter values for cytB were base frequencies of A = 0.3274, T = 0.3671, C = 0.1919, G = 0.1145; shape of gamma distribution = 0.7958; proportion of invariant sites = 0.54237. A GTR distance based neighbor-joining (NJ) tree was used as the initial topology for branch-swapping. Nodal support was evaluated by 1,000 bootstrap pseudoreplicates. For the Bayesian analyses Metropolis-coupled Markov chain Monte Carlo simulations were run with four chains (using the model selected by MrModeltest) for 5,000,000 generations. Markov chains were sampled at intervals of 100 generations to obtain 50,000 sample points. We used the default settings for the priors on topologies and the GTR + I + G model parameters. At the end of the analyses, the average standard deviations of split frequencies were 0.005692 (COI dataset) and 0.004193 (cytB dataset) and the potential scale reduction factor (PSRF) was 1, indicating that the runs had reached convergence. The sampled trees with branch lengths were used to generate 50% majority rule consensus trees, with the first 25% of the samples removed to ensure that the chain sampled a stationary portion.

Large adult females were used for shell measurements. The total number of shell whorls (WH) was counted for each specimen; and the height and width of the entire shell (SH, SW), body whorl (HBW, WBW), and aperture (AH, AW) were measured from camera lucida outline drawings (Hershler 1989). Descriptive statistics were generated using Systat for Windows 11.01 (SSI 2004). Other methods of morphological study were routine (Hershler et al. 2007).

## Results

**Figure 1.**
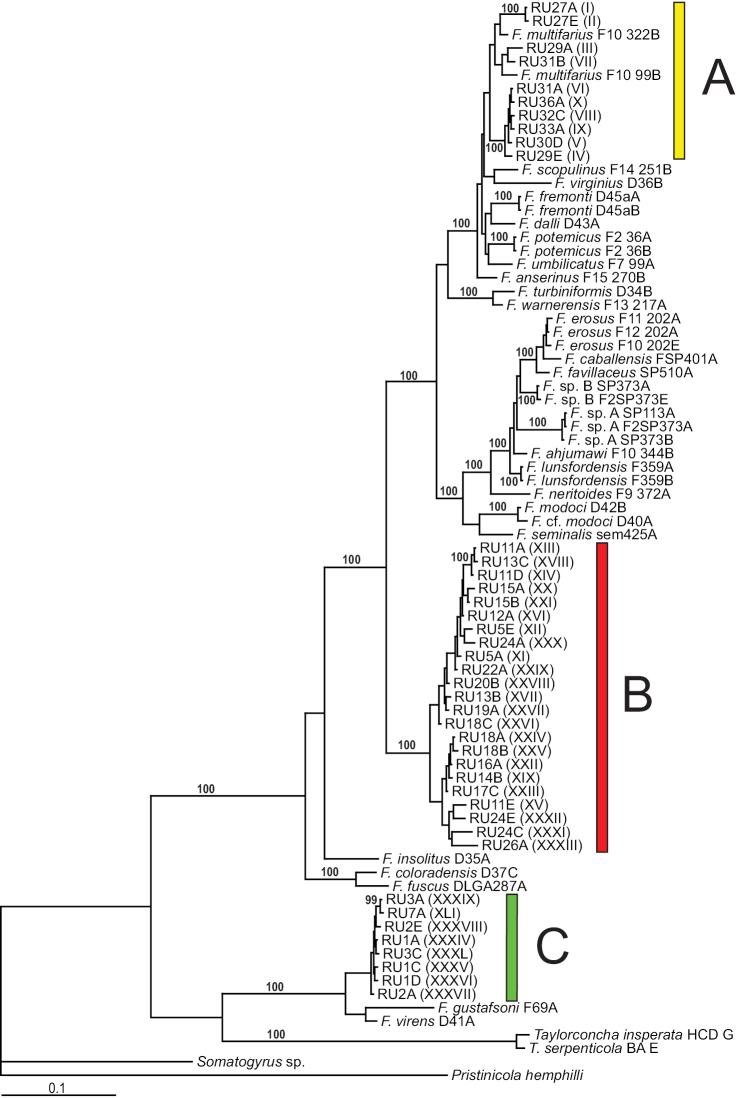
Bayesian tree based on the COI dataset. The three clades (**A–C**) containing Rogue-Umpqua haplotypes are color coded as in Figure 2. Posterior probabilities for nodes are shown when >95%. Specimen codes are from Suppl. material 1; Rogue-Umpqua haplotype codes (in parentheses) are from Suppl. material 2.

Forty-one COI and 55 cytB haplotypes were detected in the analyzed specimens from the Rogue-Umpqua basins (Suppl. material 2–3, respectively). The molecular phylogenetic analyses of both the COI and cytB datasets consistently resolved the Rogue-Umpqua haplotypes into three distinct clades. The trees generated by the three methods of phylogenetic analysis were closely similar; the Bayesian topology based on the COI dataset is shown in Figure 1.

**Figure 2.**
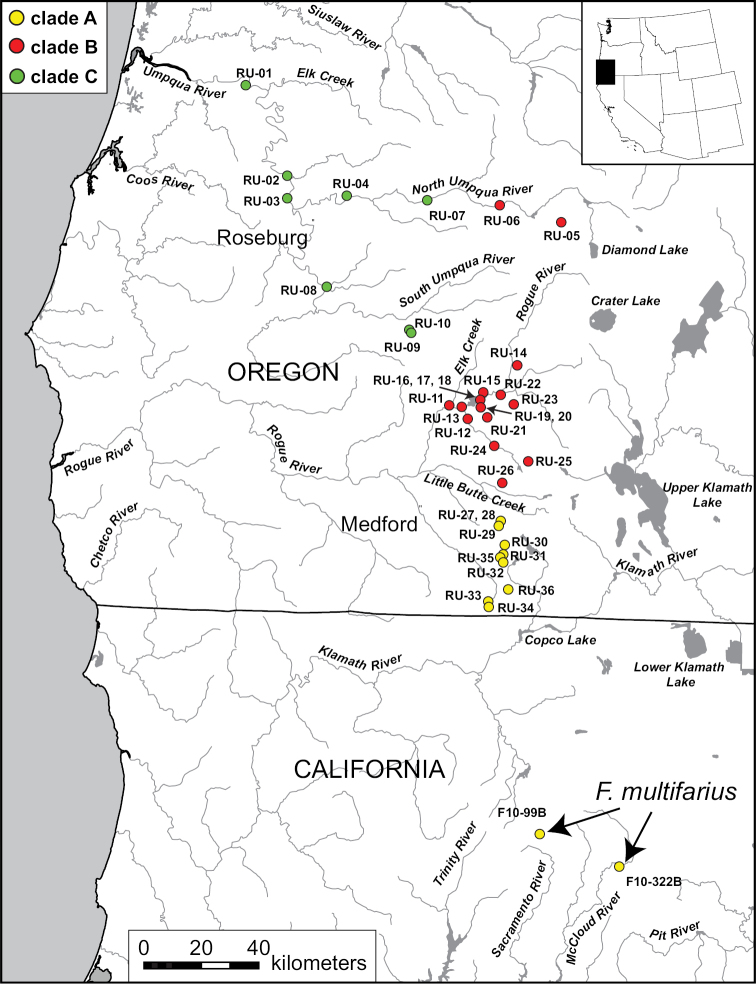
Map of southwest Oregon and northwest California showing the distribution of mtDNA clades **A–C** with color codes matching those in Fig. 1. Specimen codes are from Suppl. material 1.

Two of the clades (clades A, B, Fig. 1) contained the smaller Rogue-Umpqua pebblesnails. Clade A, which was weakly supported generally, but well supported in the cytB Bayesian topology (100% posterior probability), was composed of the haplotypes from populations in the upper Rogue River basin to the south of Little Butte Creek (Fig. 2) and the two representative sequences of *F. multifarius* Hershler, Liu, Frest & Johannes, 2012, a morphologically variable species that is distributed in the upper Sacramento River basin in northern California (Hershler et al. 2007). The divergence between the Rogue pebblesnails in clade A and all published sequences of *F. multifarius* (2.6% for COI and 3.6% for cytB, Hershler et al. 2007) was slightly greater than the variation within these two groups (2.2 and 1.4% for COI; and 3.5 and 2.4% for cytB, respectively) and falls into the range of pairwise differences among currently recognized *Fluminicola* species: 1.4–18.7% for COI and 1.6–25.7% for cytB (Hershler et al. 2007, Hershler and Liu 2012). However, we could not differentiate these geographically disjunct yet phylogenetically intermixed groups of populations based on morphologic criteria and thus are treating them as conspecific.

Clade B, which was well supported in all but the cytB Bayesian analysis, is composed of the small pebblesnails in the North Umpqua basin, and the upper Rogue River basin north of Little Butte Creek (Fig. 2). The members of this clade differ from all currently recognized *Fluminicola* species by >9.1% for both genes (the sequence divergence within the clade is *c.* 2% for both genes) and although having the generalized morphology shared by most of the smaller *Fluminicola* species, can also be distinguished from closely similar and geographically proximal *F. multifarius* by several anatomical characters. Based on the sum of this evidence we recognize clade B as a somewhat variable new species which is described below.

The third clade (C), which was moderately supported in most of the analyses and well supported in the COI ML tree (100%), contained the haplotypes detected in the large pebblesnails from the Umpqua River basin (Fig. 2). The sequence divergence within this clade was slight – 0.3% for COI and 0.6% for cytB, respectively. Clade C, in turn, formed a well-supported monophyletic group with *F. gustafsoni* and *F. virens*, and is most similar genetically to the latter, from which it differs by 3.6% for COI and 3.9% for cytB. The large pebblesnail in the Umpqua River basin is also readily differentiated morphologically from both *F. gustafsoni* and *F. virens*, and thus we recognize it as a new species which is described below.

## Systematic descriptions

### Family Lithoglyphidae Troschel, 1857

#### Genus *Fluminicola* Carpenter, 1864

##### Fluminicola multifarius

Taxon classificationAnimaliaCaenogastropodaLithoglyphidae

Hershler, Liu, Frest & Johannes, 2007

Figs 3 , 4A–D

Fluminicola multifarius . – Hershler et al. 2007: 415, 417, 419, figs. 7M, 24, 25 (Big Springs (source) at Big Springs City Park northwest of the city of Mount Shasta, south of Spring Hill, Siskiyou County, California ([UTM zone 10] 556400 E, 4575265 N, 1092 m).

###### Distribution.

Sacramento River headwater region (as far downflow as Conant), and a few sites in the upper reaches of the McCloud River drainage (Hershler et al. 2007).

###### Referred material.

OREGON. *Jackson County*. USNM 1144635, USNM 1145050, USNM 1145051, USNM 1297159, spring run on east side of BLM 37-3E-31.0, 0.24 rd. km north of BLM 38-2E-11 junction (42.2996°N, 122.5198°W), USNM 1144556, USNM 1145001, USNM 1144557, USNM 1145002, USNM 1297160, spring influenced creek at BLM 37-3E-31.0 crossing, 0.32 rd. km north of BLM 38-2E-11.0 junction (42.3001°N, 122.5198°W), USNM 1144554, USNM 1144555, USNM 1144999, USNM 1145000, USNM 1297161, spring influenced creek at crossing of BLM 38-2E-11, 1.26 rd. km east of BLM 37-3E-31.0 (42.2890°N, 122.5262°W), USNM 1144582, USNM 1144583, USNM 1144584, USNM 1144682, USNM 1145010, USNM 1145011, USNM 1145012, USNM 1297162, spring on north side of Burnt Creek Road (BLM 39-3E-21.0), 0.64 rd. km south of BLM 39-3E-32.1 junction, 1.29 km northwest of Cottonwood Glades, 3.22 rd. km south of Dead Indian Memorial Road (Jackson County 722) (42.2212°N, 122.5008°W), USNM 1144580, USNM 1144581, USNM 1144928, USNM 1297163, Cold Spring on south side of Burnt Creek Road (BLM 39-3E-21.0), 0.8 rd. km east of junction with BLM 39-3E-17.0 (42.1944°N, 122.5120°W), USNM 1144547, USNM 1144548, USNM 1144681, USNM 1144927, USNM 1144996, USNM 1144997, USNM 1297164, spring run above (north of) and below BLM 39-3E-17.0, ca. 0.56 rd. km north of BLM 39-3E-18.1 junction, northeast of Round Mountain (42.1814°N, 122.5076°W), USNM 1144566, USNM 1144567, USNM 1144909, USNM 1297165, spring on east side of (above) BLM 40-2E-35.0 at MP 0.35 off BLM 40-2E-33, east of Emigrant Creek, north of Pilot Rock, west of Porcupine Mountain (42.0438°N, 122.5612°W), USNM 1144607, USNM 1144916, spring on south side of BLM road, 0.16 km off BLM 40-2E-33, east of Emigrant Creek, north of Pilot Rock, southwest of Porcupine Mountain (42.0387°N, 122.5614°W), USNM 1144609, USNM 1144930, Sampson Creek on both sides of BLM 38-3E-18.1 (42.1869°N, 122.5167°W), USNM 1144614, USNM 1144686, USNM 1145027, USNM 1145028, spring south of Hobart Lake, west of Hobart Bluff, 0.21 km east of BLM 39-3E-32.3, 0.64 km north of Hobart Peak (42.0933°N, 122.4799°W).

###### Comparative material.

CALIFORNIA. *Siskiyou County*. USNM 1070753, Big Springs (source) at Big Springs City Park northwest of the city of Mount Shasta, south of Spring Hill, USNM 1020771, Bundoora Spring, west of access road off FS40N44.

###### Remarks.

**Figure 3.**
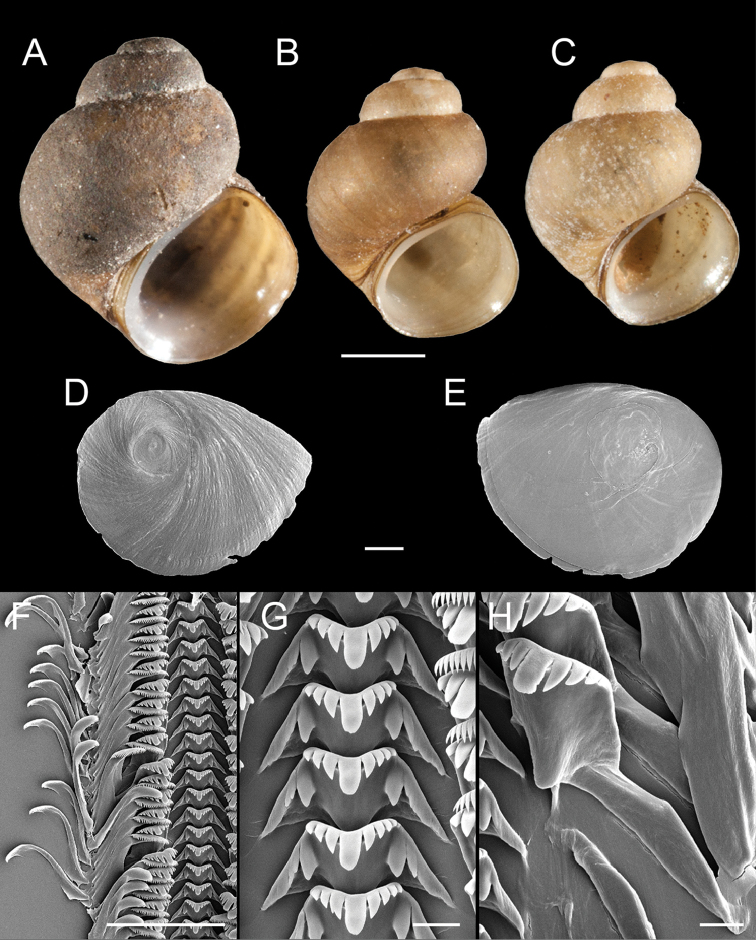
Shells, opercula and radula, *F. multifarius*. **A** Shell, USNM 1144567 **B** Shell, USNM 1144997 **C** Shell, USNM 1145051 **D, E** Opercula (outer, inner sides), USNM 1144567 **F** Portion of radular ribbon, USNM 1144567 **G** Central teeth, USNM 1144567 **H** Lateral teeth, USNM 1144567. Scale bars: **A–B** 1.0 mm; **D–E** 250 µm; **F**, 100 µm; **G–H** 10 µm.

The newly reported populations closely conform to the original description of *F. multifarius* in all respects aside from a slightly larger maximum shell height (5.02 vs. 4.64 mm). Representative Rogue basin specimens are illustrated in Figures 3A–C (shells), 3D–E (opercula), 3F–H (radula) and 4A, B, D (reproductive anatomy). Ten COI and cytB haplotypes were detected in the Rogue basin populations (Suppl. material 2–3, respectively).

The new records detailed herein extend the range of *F. multifarius* about 80 km northward from the Sacramento River headwaters (Fig. 2). It is not known whether *F. multifarius* is also distributed in the intervening Klamath River basin; the pebblesnail fauna of this large watershed is currently undescribed. Populations of *F. multifarius* in the Rogue basin were referred to as the Chinquapin pebblesnail, Emigrant pebblesnail, Keene Creek pebblesnail, Little Butte pebblesnail, and Pilot Rock pebblesnail by Frest and Johannes (2000, 2004, 2005).

**Figure 4.**
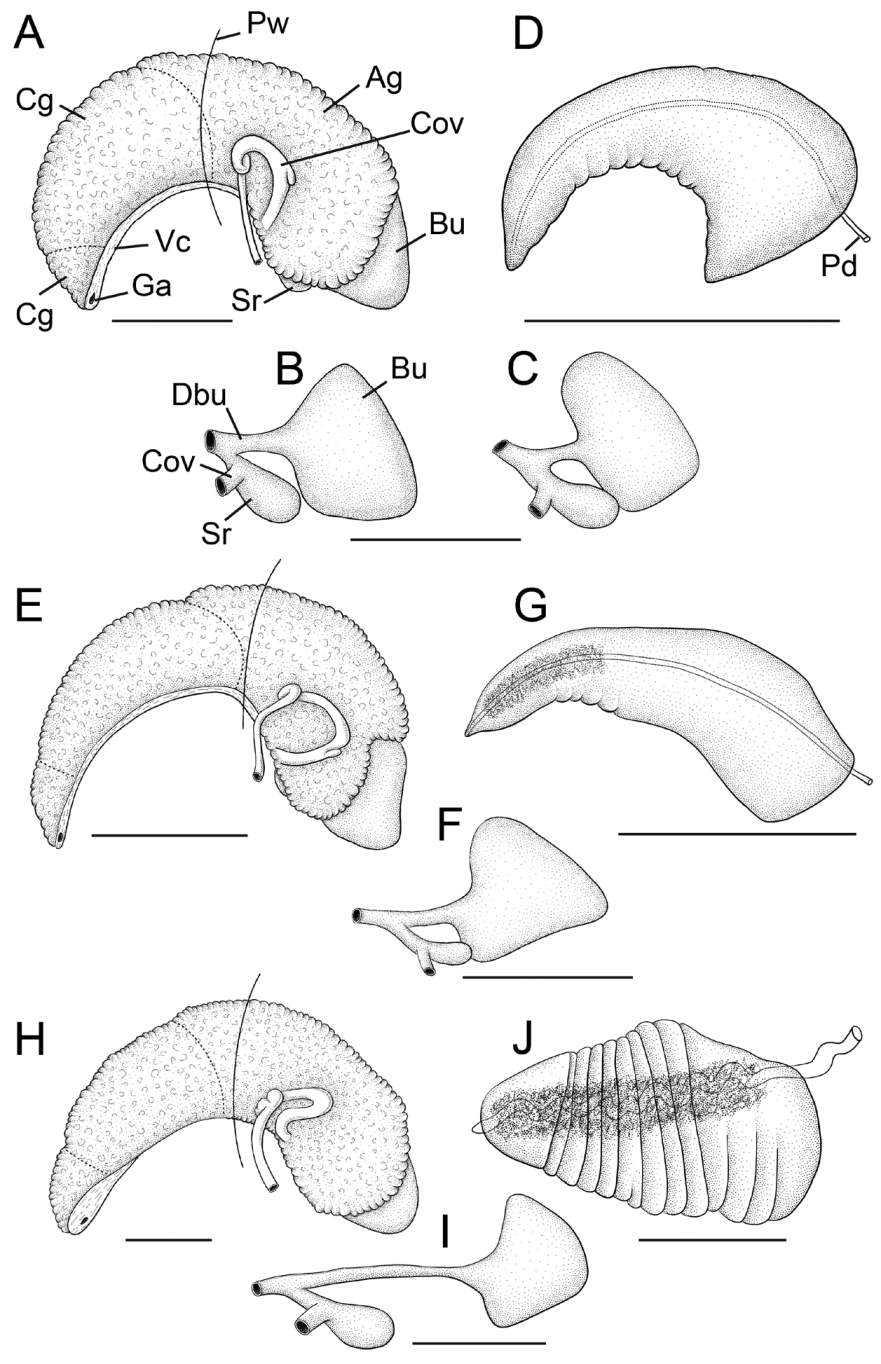
Reproductive anatomy. **A–D** *F. multifarius* **A, B, D** USNM 1144567, **C** USNM 1070753 **E–G** *F. fresti*, sp.n., USNM 1422223 **H–J** *F. umpquaensis*, sp. n., USNM 1144736. **A, E, G** Female glandular oviduct and associated structures (viewed from left side) **B, C, F, I** Bursa copulatrix and seminal receptacle, **D, G, J** Penis, dorsal surface. The bursa copulatrix and seminal receptacle of type material of *F. multifarius* (**C**), which was not illustrated by Hershler et al. (2007), is shown for comparative purposes. Scale bars, 500 µm. **Ag** albumen gland **Bu** bursa copulatrix **Cg** capsule gland **Cov** coiled oviduct **Dsr** seminal receptacle duct **Ga** genital aperture **Pd** penial duct **Pw** posterior wall of pallial cavity **Sr** seminal receptacle **Vc** ventral channel of capsule gland.

##### Fluminicola fresti

Taxon classificationAnimaliaCaenogastropodaLithoglyphidae

Hershler, Liu & Hubbart sp. n.

http://zoobank.org/75362D82-9786-4423-9435-CD99A795C050

Figs 4E–G , 5

###### Types.

Holotype, USNM 1144376, diversion from Big Butte Springs through Butte Falls Hatchery, just south of Butte Falls-Fish Lake Road (Jackson County 321) and 0.16 km west of Butte Falls-Prospect Road (Jackson County 922), Jackson County, Oregon, 42.5389°N, 122.5551°W, 10/22/1994, Terrence J. Frest and Edward J. Johannes. Paratypes, USNM 1422223 (a large series of dry shells and alcohol-preserved specimens), from same lot.

###### Referred material.

OREGON. *Douglas County*: USNM 1297126, Trap Creek at crossing of FS4788, 0.48 km south of its confluence with the Clearwater River (43.2431°N, 122.2886°W). *Jackson County*. USNM 1144448, USNM 1297142, Rogue River on north side at Rogue Elk County Park, east of Rogue Elk, south of OR62 (42.6618°N 122.7524°W), USNM 1144532, USNM 1297143, Vine Creek on east side of McNeil Road (42.6270°N, 122.6722°W), USNM 1144525, USNM 1297144, Rogue River, upriver from Casey State Park boat ramp, west of McLeod (42.6591°N, 122.6993°W), USNM 1297145, Mill Creek at Mill Creek Campground off OR62 on FS030, ca. 5.6 km northeast of Prospect (42.7937°N, 122.4679°W), USNM 1144901, USNM 1145101, USNM 1145102, USNM 1297146, Evergreen Spring above Lost Creek Lake (42.7026°N, 122.6090°W), USNM 1297147, spring outflow, at foot bridge crossing, Joseph Stewart State Park (42.6810°N, 122.6216°W), USNM 1297149, spring outflow, first crossing above foot bridge, Joseph Stewart State Park (42.6811°N, 122.6204°W), USNM 1297150, Lost Creek at BLM 34-2E-8 (Medco A Road) crossing (north side), south of Joseph Stewart State Park (42.6570°N, 122.6152°W), USNM 1144528, USNM 1297152, Middle Fork Lost Creek at BLM 34-2E-8 (Medco A Road) crossing (east side), south of Joseph Stewart State Park (42.6571°N, 122.6150°W), USNM 1297153, Clark Creek collected east of BLM 34-2E-9.02 crossing off Clark Creek Road (BLM 34-2E-7) (42.6305°N, 122.5865°W), USNM 1144526, USNM 1297154, spring below (northeast of) BLM 33-2E-13.02, 0.32 rd.km off of BLM 33-2E-13.01, west of Smith Creek (42.7015°N, 122.5347°W), USNM 1144531, USNM 1297155, southern-most spring of three on west side of BLM 33-3E-28.01, 0.8 rd. km north of BLM 33-3E-34 junction, east of Reinecke Burn (42.6733°N, 122.4749°W), USNM 1297156, diversion from Big Butte Springs through Butte Falls Hatchery, USNM 1144375, USNM 1297157, Whiskey Spring at Whiskey Spring Campground on FS100 off FS37 (42.4949°N, 122.4151°W), USNM 1144533, USNM 1144534, USNM 1297158, spring in Wasson Canyon on south side of Wasson Canyon Creek above (east of) BLM 36-2E-19.2 off of BLM 36-2E-26 (42.4240°N, 122.5206°W).

###### Diagnosis.

A small to medium-sized *Fluminicola* (2.3–5.5 mm shell height) having a trochoidal to ovate-conic shell and small, gently tapered penis. Differs from closely similar and geographically proximal *F. multifarius* in the hooked shape of the anterior end of the osphradium, larger number of ctenidial (gill) filaments, smaller seminal receptacle, and in its mtDNA sequences.

###### Description.

**Figure 5.**
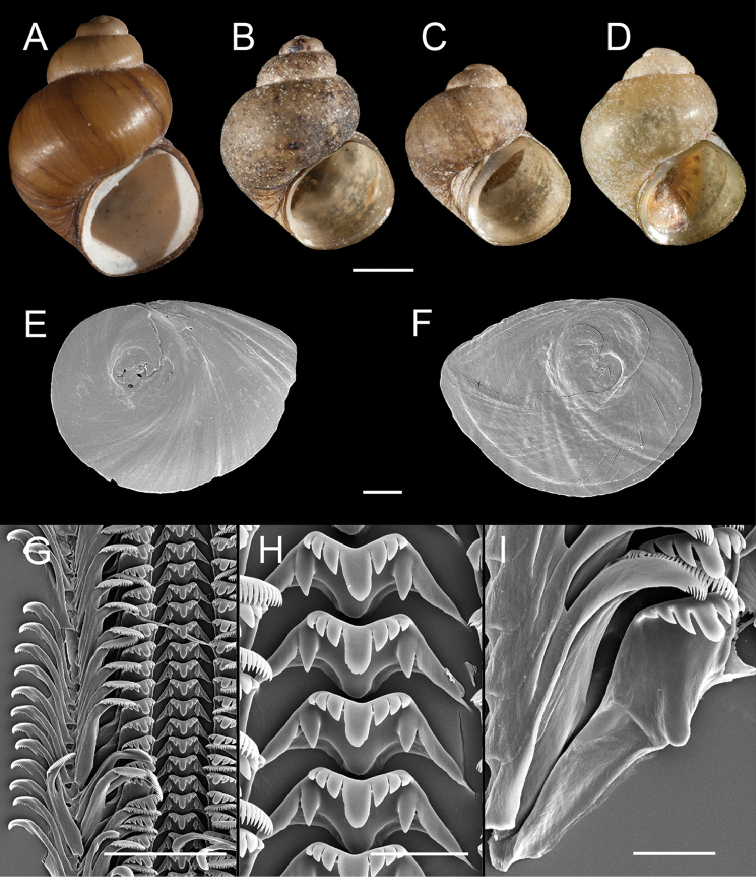
Shells, opercula and radula, *F. fresti*, sp. n. **A** Holotype, USNM 1144376 **B, C** Sympatric ovate-conic and trochiform shell morphs, respectively, USNM 1297149 **D** Shell, USNM 1297155 **E, F** Opercula (outer, inner sides), USNM 1422223 **G** Portion of radular ribbon, USNM 1422223 **H** Central teeth, USNM 1422223 **I** Lateral teeth, USNM 1422223. Scale bars: **A–D** 1.0 mm; **E–F** 250 µm; **G** 100 µm; **H–I** 20 µm.

Shell (Fig. 5A–D) trochoidal to narrow-conic, whorls 3.5–4.0. Teleoconch whorls medium convex, sometimes weakly shouldered. Aperture ovate, slightly angled above; inner lip complete, variably thickened and reflected, sometimes forming a rather wide parietal-columellar shelf that sometimes covers the umbilical region. Outer lip thin, prosocline. Umbilicus very small or absent, umbilical region sometimes excavated. Shell white, periostracum brown, sometimes covered with thick black deposits. Shell measurements and whorl count data are summarized in Table 1.

**Table 1.** Shell parameters for *F. fresti*. Measurements are in mm.

	WH	SH	SW	HBW	WBW	AH	AW
Holotype, USNM 1144376							
	4.00	4.36	3.12	3.55	2.76	2.20	2.03
Paratypes, USNM 1422223 (*N* = 17)							
Mean	4.03	4.37	3.42	3.61	2.82	2.28	2.09
S.D.	0.17	0.41	0.30	0.31	0.23	0.22	0.19
Range	3.75–4.25	3.93–5.46	3.07–4.18	3.18–4.31	2.58–3.37	1.92–2.77	1.77–2.48

Operculum (Fig. 5E–F) as for genus; muscle attachment margin thickened on inner side. Radula (Fig. 5G–I) as for genus; dorsal edge of central teeth concave, lateral cusps two–five, basal cusp one–two. Lateral teeth having two–three cusps on inner side and three–four cusps on outer side; length of outer wing 175–185% length of cutting edge. Inner marginal teeth with 23–31 cusps, outer marginal teeth with 27–40 cusps. Radula data are from USNM 1422223, USNM 1144426.

Snout, cephalic tentacles, pallial roof, visceral coil usually medium pigmented (brown); foot varying from near pale to medium pigmented along anterior edges. Distal section of penis having dense core of internal black pigment. Ctenidial filaments 21–24 (*N* = 5), lateral surfaces smooth. Anterior end of osphradium distinctly hooked (not illustrated). Glandular oviduct and associated structures shown in Figure 4E–F. Coiled oviduct circular, proximal arm kinked, posterior arm sometimes having small pouch containing sperm. Bursa copulatrix large, reniform, partly overlapped by albumen gland. Bursal duct slightly shorter than bursa copulatrix, narrow. Seminal receptacle small, sac-like, almost completely overlapped by albumen gland. Albumen gland having short pallial component. Capsule gland longer than albumen gland, composed of two glandular zones. Genital aperture a small, sub-terminal pore. Penis (Fig. 4G) small, slightly curved, gently tapering, distal section abruptly narrowing to small pointed tip. Medial section having a few weak folds along inner edge. Penial duct near centrally positioned, straight, narrow.

###### Etymology.

This species name is a patronym (in the genitive singular) honoring recently deceased malacologist Terrence Frest for his many contributions to the documentation of molluscan biodiversity in the northwestern United States.

###### Distribution.

*Fluminicola fresti* is distributed in spring-fed habitats in the North Umpqua River drainage and in the Rogue River basin north of Little Butte Creek.

###### Remarks.

As noted above, the shells of *F. fresti* vary in overall shape and in the width of the inner apertural lip. Although this variation is generally continuous in the material that we examined, two rather distinct forms – ovate-conic with a narrow inner apertural lip (Fig. 5B) and trochoidal with a wide parietal-columellar shelf (Fig. 5C) – can be identified in one of the springs in Joseph Stewart State Park. We sequenced specimens from this locality and found that the two shell forms (samples RU18 and RU17, respectively) differed by 0.7% for COI and 1.3% for cytB, which was less than the variation within these two groups (0.2 and 1.2% for COI; and 0.2 and 1.5% for cytB, respectively). The scant genetic differentiation of the two shell forms when in sympatry provides additional evidence that they are conspecific. Additional studies incorporating rapidly evolving nuclear markers (e.g., microsatellites) may help sift through the possible explanations for the interesting variation in the shells of this species (e.g., ecophenotypic plasticity, incipient speciation). Twenty-three COI haplotypes and 34 cytB haplotypes were detected in the sequenced specimens of *F. fresti* (Suppl. material 2–3, respectively).

Populations of *F. fresti* were referred to as the Beaverdam pebblesnail, Camp Creek pebblesnail, Clark pebblesnail, Evergreen pebblesnail, Rogue pebblesnail, Stewart pebblesnail, and Umpqua pebblesnail by Frest and Johannes (1999, 2000, 2004, 2005). In order to avoid confusion, we suggest that “Frest’s pebblesnail” be used as the common name for *F. fresti*.

##### Fluminicola umpquaensis

Taxon classificationAnimaliaCaenogastropodaLithoglyphidae

Hershler, Liu & Hubbart sp. n.

http://zoobank.org/7EEF6D2F-5CAB-4CD2-A41C-E1EB8D556EBD

Figs 4H–I , 6

Fluminicola virens .–Pilsbry 1899: 122-123 (in part). Henderson 1929: 169 (in part). Burch and Tottenham 1980: 102 (in part).

###### Types.

Holotype, USNM 1144535, Umpqua River at Bunch Bar Access (County), west of OR38 (Umpqua Highway), Douglas County, Oregon, 43.6462°N, 123.6670°W, 9/27/1998, Terrence J. Frest and Edward J. Johannes. Paratypes, USNM 1422224 (a large series of dry shells and alcohol-preserved specimens), from same lot.

###### Referred material.

OREGON. *Jackson County*. USNM 1296909, Umpqua River at Bunch Bar Access, USNM 1296910, Umpqua River at Umpqua County Landing upriver of Sutherlin-Umpqua Road Bridge (Douglas County 9) on east side, north of the mouth of Calapooya Creek, opposite of the town of Umpqua, RM 102.7 (43.3661°N, 123.4677°W), USNM 1296911, east side of Umpqua River at Cleveland Rapids, Cleveland Rapids Park, opposite and northeast of Cleveland (43.2966°N, 123.4705°W), USNM 1144361, USNM 126912, east side of North Umpqua River near boat ramp on west side of Whistlers Bend County Park (43.3101°N, 123.2168°W), USNM 1297138, bedrock shelf and bar on west side of North Umpqua River, ca. 0.48 km southwest of mouth of Swamp Creek (43.3015°N, 122.8692°W), USNM 1297139, Myrtle Creek just upstream of OR99 (Myrtle Creek Highway) bridge (43.0237°N, 123.2890°W), USNM 1297140, Elk Creek just below confluence of Drew Creek (42.8907°N, 122.9227°W), USNM 1144544, USNM 1297141, Elk Creek just south of bridge of Callahan Creek Road (FS3230), west and below Tiller Trail Highway (Douglas Co. 1, OR227) (42.8973°N, 122.9308°W), USNM 1144751, Pass Creek at site of Comstock in Pass Creek County Park, west side of I-5 off exit 164 (43.7186°N, 123.2076°W), USNM 1144360, USNM 1144656, south side of North Umpqua River at John P. Amacher Park, under and down river of railroad bridge west of I-5 and Winchester (43.2796°N, 123.3557°W), USNM 1144726, west side of North Umpqua River at and 0.32 km upstream of Chris Hestness Landing (43.2843°N, 123.1674°W), USNM 1144730, Umpqua River at Umpqua County Landing upriver of Sutherlin-Umpqua Road Bridge (Douglas County 9) on east side, north of the mouth of Calapooya Creek, opposite of Umpqua (43.3643°N, 123.4665°W), USNM 1144736, east side of Umpqua River at Cleveland Rapids, Cleveland Rapids Park, opposite and northeast of Cleveland (43.2972°N, 123.4686°W), USNM 1144748, Myrtle Creek just upstream of OR99 (Myrtle Creek Highway) bridge (43.0220°N, 123.2871°W), USNM 1144371, USNM 1144948, west side of South Umpqua River, east of I-5 and north of OR99 (Myrtle Creek Highway) Bridge, west of town of Myrtle Creek (43.0238°N, 123.2954°W), USNM 1144372, South Myrtle Creek, ca. 2.82 km from junction with North Myrtle Creek, below Days Creek Road (43.0176°N, 123.2666°W), USNM 1144473, north side of South Umpqua River just upstream (east) of mouth of Dumont Creek at Dumont Creek Campground (43.0337°N, 122.8010°W), USNM 1144545, Umpqua River on south side, Scottsburg County Park boat ramp (upstream side), southwest of Scottsburg (43.6480°N, 123.8377°W), USNM 1144546, USNM 1144995, Cow Creek at mouth of Salt Creek, west of Cow Creek Road (Douglas Co. 39), east of Byers (42.9240°N, 123.4897°W), BellMNH uncat., North Umpqua River at fish traps above Ideyld (43.3220°N, 123.0248°W), BellMNH uncat., North Umpqua River, Glide (43.302°N, 123.101°W), BellMNH uncat., forks of Umpqua River below Roseburg, BellMNH uncat., North Umpqua River at Winchester (43.282°N, 123.355°W), BellMNH uncat., North Umpqua River near Glide, BellMNH uncat., South Umpqua River near Canyonsville, BellMNH uncat., Umpqua River, Basket Point, BellMNH uncat., Umpqua River, Elkton (43.635°N, 123.570°W).

###### Diagnosis.

A large *Fluminicola* (maximum shell height, 9.5 mm) having a conical shell with eroded spire and a broad, little tapered penis. Differs from closely related *F. gustafsoni* and *F. virens* in its reniform-shaped bursa copulatrix and in its mtDNA sequences. Further differentiated from *F. gustafsoni* by its more elongate shell, longer outer wing of the lateral radular teeth, and smaller seminal receptacle; and from *F. virens* by its more convex shell whorls and longer bursal duct.

###### Description.

**Figure 6.**
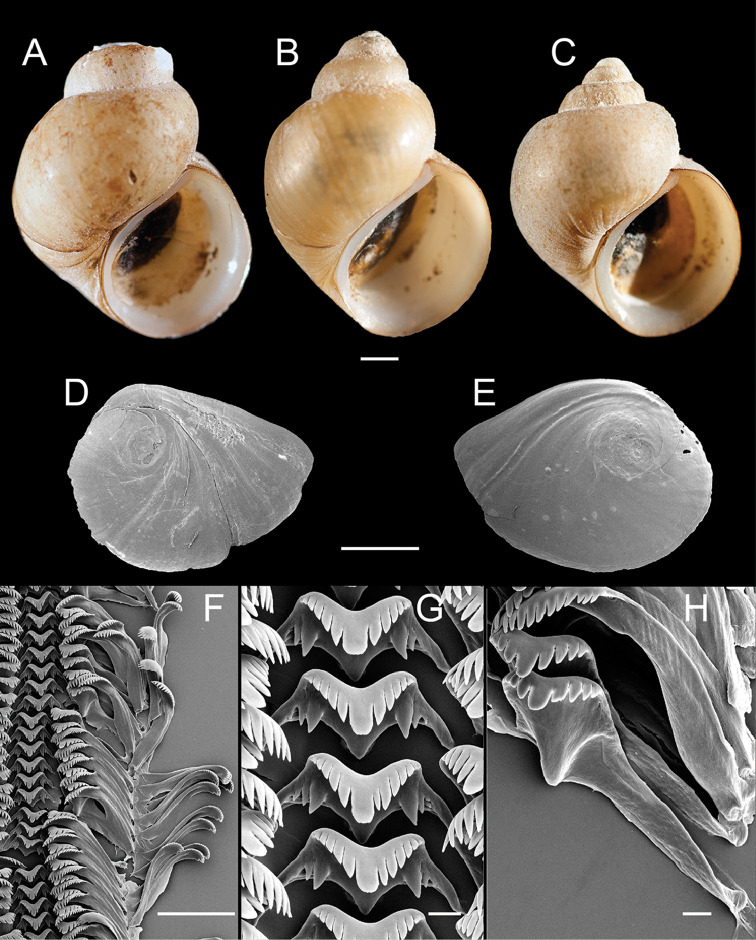
Shells, opercula and radula, *F. umpquaensis*, sp. n. **A** Holotype, USNM 1144535 **B** Shell, USNM 1144736 **C** Shell, USNM 1144371 **D, E** Opercula (outer, inner sides), USNM 114535 **F** Portion of radular ribbon, USNM 1422224 **G** Central teeth, USNM 1422224 **H** Lateral teeth, USNM 1422224. Scale bars: **A–E**, 1.0 mm; **F**, 100 µm; **G–H**, 10 µm.

Shell (Fig. 6A–C) conical, spire usually eroded, whorls >3.5. Teleoconch whorls medium convex, narrowly shouldered. Aperture ovate, slightly angled above; inner lip complete, thickened. Outer lip thin, prosocline. Umbilicus absent. Shell white, periostracum tan or olive. Shell measurements and whorl count data are summarized in Table 2.

**Table 2.** Shell parameters for *F. umpquaensis*. Measurements are in mm.

	SH	SW	HBW	WBW	AH	AW
Holotype, USNM 1144535						
	7.77	5.97	6.64	4.93	4.39	3.88
Paratypes, USNM 1422224 (*N* = 10)						
Mean	7.37	5.71	6.57	4.91	4.24	3.80
S.D.	0.36	0.35	0.28	0.33	0.22	0.22
Range	6.89–7.96	5.37–6.48	6.21–7.25	4.59–5.73	3.89–4.59	3.56–4.29

Operculum (Fig. 6D–E) as for genus; inner side smooth. Radula (Fig. 6F–H) as for genus; dorsal edge of central teeth concave, lateral cusps four–five, basal cusp two–four. Lateral teeth having three–six cusps on inner side and four–six cusps on outer side; length of outer wing 180–200% length of cutting edge. Inner marginal teeth with 13–18 cusps, outer marginal teeth with 11–17 cusps. Radula data are from USNM 1422224.

Head-foot rather lightly pigmented, cephalic tentacles with central brown longitudinal stripe along length. Pallial roof, visceral coil dark brown, almost black. Penis having dense core of internal black pigment along penial duct. Ctenidial filaments 34–36 (*N* = 5), lateral surfaces ridged. Glandular oviduct and associated structures shown in Figure 4H–I. Coiled oviduct vertical or posterior-oblique, proximal arm kinked. Bursa copulatrix medium-sized, reniform, largely overlapped by albumen gland. Bursal duct about twice as long as bursa copulatrix, very narrow. Seminal receptacle small, sac-like, completely overlapped by albumen gland. Albumen gland having short pallial component. Capsule gland slightly shorter than albumen gland, composed of two glandular zones. Genital aperture a small, sub-terminal pore. Penis (Fig. 4J) large, straight, broad, little tapered, distally rounded, deeply folded along most of length. Penial duct near centrally positioned, rather wide, undulating along entire length, opening through small terminal papilla.

###### Etymology.

The species name is an adjectival geographic epithet referring to the distribution of this pebblesnail in the Umpqua River basin.

###### Distribution.

*Fluminicola umpquaensis* is widely ranging in the Umpqua River basin, and is distributed in riverine habitats as well as springs and streams.

###### Remarks.

As mentioned above, the smaller of the two divergent *Fluminicola* clades (containing *F. gustafsoni* and *F. virens*) was previously confined to the Columbia River basin (Hershler and Liu 2012). *Fluminicola umpquaensis* extends the geographic range of this lineage >200 km southward from the lower Columbia River. Eight COI haplotypes and 11 cytB haplotypes were detected in the sequenced specimens of *F. umpquaensis* (Suppl. material 2–3, respectively).

Populations identified herein as *F. umpquaensis* were referred to as the Jade pebblesnail by Frest and Johannes (2000:182). We propose that this common name continue to be applied to this species.

## Supplementary Material

XML Treatment for Fluminicola multifarius

XML Treatment for Fluminicola fresti

XML Treatment for Fluminicola umpquaensis

## Supplementary materials

Supplementary material 1 Sample codes, locality details (voucher numbers for newly sequenced specimens in parentheses), and GenBank accession numbers for COI and cytB sequences Data type: Specimen data This dataset is made available under the Open Database License (http://opendatacommons.org/licenses/odbl/1.0/). The Open Database License (ODbL) is a license agreement intended to allow users to freely share, modify, and use this Dataset while maintaining this same freedom for others, provided that the original source and author(s) are credited.Robert Hershler, Hsiu-Ping Liu, Niko Hubbart

Supplementary material 2 Distribution of COI haplotypes Data type: Genetic data Explanation note: Exemplars used in the phylogenetic analyses are in parentheses. This dataset is made available under the Open Database License (http://opendatacommons.org/licenses/odbl/1.0/). The Open Database License (ODbL) is a license agreement intended to allow users to freely share, modify, and use this Dataset while maintaining this same freedom for others, provided that the original source and author(s) are credited.Robert Hershler, Hsiu-Ping Liu, Niko Hubbart

Supplementary material 3 Distribution of cytB haplotypes Data type: Genetic data Explanation note: Exemplars used in the phylogenetic analyses are in parentheses. This dataset is made available under the Open Database License (http://opendatacommons.org/licenses/odbl/1.0/). The Open Database License (ODbL) is a license agreement intended to allow users to freely share, modify, and use this Dataset while maintaining this same freedom for others, provided that the original source and author(s) are credited.Robert Hershler, Hsiu-Ping Liu, Niko Hubbart
